# Development and Application of a Fully Automated Chemiluminescence Enzyme Immunoassay for the Detection of Antibodies Against Porcine Circovirus 3 Cap

**DOI:** 10.3390/v16121925

**Published:** 2024-12-17

**Authors:** Lei Wang, Duan Li, Daoping Zeng, Xiaomin Wang, Yanlin Liu, Guoliang Peng, Zheng Xu, Changxu Song

**Affiliations:** 1 College of Life Sciences, Longyan University, Longyan 364012, China; wangllei2024@163.com; 2 State Key Laboratory of Swine and Poultry Breeding Industry, National Engineering Center for Swine Breeding Industry, College of Animal Science, South China Agricultural University, Guangzhou 510642, China; lduan202412@163.com (D.L.); 19861927085@163.com (X.W.); yll395070943@163.com (Y.L.); 3 Wens Foodstuff Group Co., Ltd., Yunfu 527400, China; zengdaoping2000@163.com; 4 Biaoyun Biotechnology Co., Ltd., Yunfu 527400, China; 5 Henry Fok School of Biology and Agriculture, Shaoguan University, Shaoguan 512005, China; pengguoliang168@163.com

**Keywords:** porcine circovirus 3, cap, chemiluminescence enzyme immunoassay, antibody detection

## Abstract

Porcine circovirus 3 (PCV3) is a small non-enveloped circovirus associated with porcine dermatitis and nephropathy syndrome (PDNS). It has occurred worldwide and poses a serious threat to the pig industry. However, there is no commercially available vaccine. PCV3 capsid protein (Cap) is an ideal antigen candidate for serodiagnosis. Here, a novel fully automated chemiluminescence enzyme immunoassay (CLEIA) was developed to detect antibodies (Abs) to Cap in porcine serum. Recombinant PCV3 Cap, self-assembled into virus-like particles (VLPs), was produced using baculovirus and coupled to magnetic particles (Cap-MPs) as carriers. Combined with an alkaline phosphatase (AP)–adamantane (AMPPD) system, Cap-Abs can be rapidly measured on a fully automated chemiluminescence analyzer. Under optimal conditions, a cut-off value of 31,508 was determined, with a diagnostic sensitivity of 96.8% and specificity of 97.3%. No cross-reactivity was observed with PCV1 and PCV2 and other common porcine pathogens, and both intra-assay and inter-assay coefficients were less than 5% and 10%, respectively. Prepared Cap-MPs can be stored at 4 °C for more than 6 months. Importantly, this CLEIA had a good agreement of 95.19% with the commercially available kit, demonstrating excellent analytical sensitivity and significantly reduced operating time and labor. A serological survey was then conducted, and showed that PCV3 continues to spread widely in South China. In conclusion, our CLEIA provides time and labor-saving, and a reliable tool for PCV3 epidemiological surveillance.

## 1. Introduction

Porcine circovirus (PCV) is a small, non-enveloped, single-stranded DNA virus. Four species of PCV have been formally accepted, including PCV1, PCV2, PCV3, and PCV4. PCV3 was first reported in 2016 on a farm in North Carolina (USA) [1,2]. However, according to several retrospective studies, it has been circulating in pig farms for almost half a century [3]. Moreover, a large number of reports demonstrate that PCV3 usually co-infects with other pathogens in pigs such as PCV2, porcine epidemic diarrhea virus (PEDV), porcine parvovirus 7 (PPV7), classical swine fever virus (CSFV), and porcine reproductive and respiratory syndrome virus (PRRSV) [4,5,6]. Astonishingly, transmission of PCV3 to baboons was also observed [7]. It is generally considered to be pathogenic and associated with porcine dermatitis and nephropathy syndrome (PDNS), reproductive failure, and respiratory disease in pigs of all ages, similar to PCV2 [8]. Therefore, PCV3 could pose a real threat to the pig industry. Serological tests would be useful to support vaccine development and epidemiological surveillance of PCV3. Several indirect enzyme-linked immunosorbent assays (ELISAs) have been developed [9]. However, ELISA is time-consuming and labor intensive. Given the increasing cost of labor, the aim of this study is to develop a simple and rapid method for the detection of antibodies against PCV3.

PCV3 has a similar morphology to other PCVs, and its genome consists of three major open reading frames (ORFs), namely ORF1, ORF2, and ORF3. ORF1 encodes the replicase protein (Rep), ORF2 encodes the capsid protein (Cap), and ORF3 encodes a protein of unknown function [10]. Phylogenetic analyses revealed a 24% amino acid sequence homology of Cap between PCV1 and PCV3, and 26–37% between PCV2 and PCV3 [11]. PCV3 Cap can self-assemble into virus-like particles (VLPs) [12]. This makes it an ideal target for serological diagnosis and vaccine development. Conventional ELISA has been used to detect antibodies to PCV3, and there is no serological cross-reactivity between PCV3, PCV1, and PCV2 Cap [9].

Chemiluminescence immunoassay involves highly selective immunoassay and extremely sensitive chemiluminescence analysis that can provide autoantibody detection. It can be divided into three categories according to the chemiluminescence reaction, namely chemiluminescence immunoassays (CLIA), chemiluminescence enzyme immunoassays (CLEIA), and electrochemiluminescence immunoassays (ECL) [13]. CLEIA is involved in the enzyme-catalyzed chemiluminescence reaction, typically horse radish peroxidase (HRP) or alkaline phosphatase (AP). Representative chemiluminescence reaction systems are HRP-H_2_O_2_-Luminol and AP-3-(2′-spiroadamantyl)-4-methoxy-4-(3″-phosphoryloxy)-phenyl-1,2-dioxetane (AMPPD) [14]. Of these, AMPPD is more stable (half-life can reach 74 years) with low non-enzymatic hydrolysis and luminescence background. AP marked in CLEIA was widely employed in enzyme-linked immunoassay for environment, food, infectious diseases, and so on [15,16,17].

In this study, a novel automated CLEIA was developed comprising Cap-coupled magnetic particles (MPs) and the AP-conjugated secondary antibody and its substrate AMPPD. Subsequently, PCV3 Cap-Abs can be measured simply and rapidly. Their performance was characterized. The prevalence of PCV3 infection was also investigated. These results demonstrated that the developed CLEIA is an automated and reliable method, with unique advantages of high sensitivity and specificity, as well as time and labor-saving, for the detection of antibodies against PCV3.

## 2. Results

### 2.1. Preparation of PCV3 Cap

**Figure 1:**
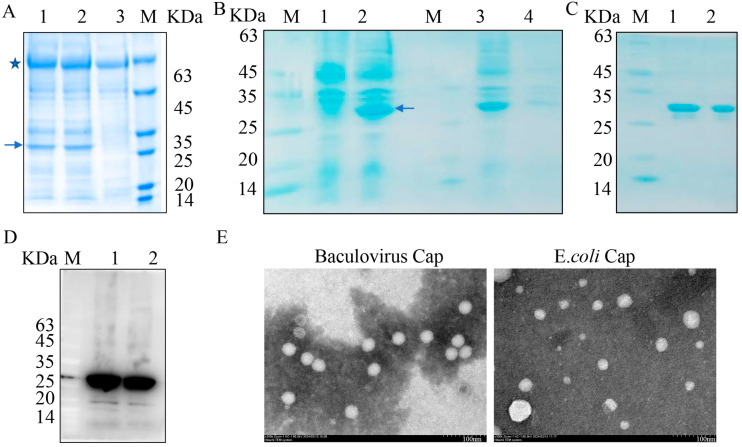
Expression and purification of recombinant PCV3 Cap. (**A**) SDS-PAGE analysis of expression of recombinant Cap. 1,2 cell lysis forms cells infected with recombinant baculovirus, 3 cell lysis forms control, M protein marker. The blue arrow indicates expressed Cap, and the blue pentagram indicates bovine serum albumin (BSA). (**B**) SDS-PAGE analysis of Cap expression in *E. coli*, 1 cell lysis forms *E. coli* uninduced, 2 cell lysis forms *E. coli* induced by IPTG, 3 lysed supernatant, 4 precipitation, M protein marker. The blue arrow indicates expressed Cap. (**C**) Purification of recombinant Cap, 1. Cap from Sf9 cells, 2. Cap from *E. coli*., M protein marker. (**D**) IB analysis of recombinant Cap using anti-PCV3 Cap monoclonal antibody, 1. Cap from Sf9 cells, 2. Cap from *E. coli*., M protein marker. (**E**) TEM images of purified Cap.

Two different systems were used to produce recombinant PCV3 Cap. As shown in Figure 1A,B, specific bands (marked by blue arrows) with an approximate molecular weight (MW) between 25 and 35 kDa were observed by sodium dodecyl sulfate polyacrylamide gel electrophoresis (SDS-PAGE) in agreement with theoretical MW, indicating that recombinant Cap was successfully expressed in insect cells and *E. coli*, respectively. In addition, Cap produced in *E. coli* was predominantly expressed in soluble form. Soluble recombinant Cap was then purified and coomassie blue staining showed a clear band for recombinant protein obtained from insect cells and *E. coli* (Figure 1C). The purified Cap was further identified by immunoblotting (IB), followed by transmission electron microscopy (TEM). The result of IB showed that recombinant Cap had good reactivity (Figure 1D). While TEM images revealed that purified Cap could self-assemble into VLPs, in particular, VLPs derived from baculovirus had a uniform conformation and nano size of ~20 nm, whereas VLPs derived from *E. coli* showed different sizes ranging from 10 to 80 nm (Figure 1E). Therefore, baculovirus-expressed Cap is an ideal antigen for subsequent immunoassay.

### 2.2. Characterization of Cap-MPs

**Figure 2:**
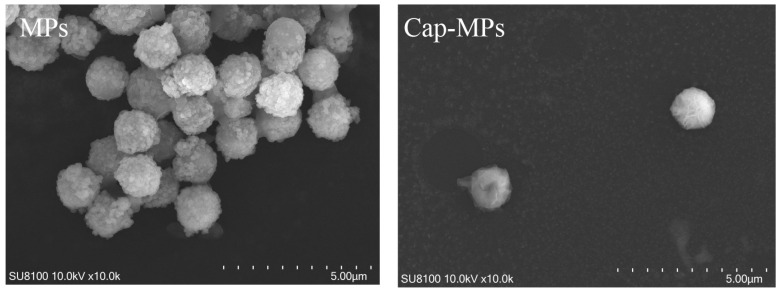
SEM images of MPs and prepared Cap-MPs. Scale bar: 5.0 μm.

The morphological change of the prepared Cap-MPs was imaged by scanning electron microscopy (SEM). As shown in Figure 2, the blank particles had a porous surface, whereas the particles of Cap-MPs were coated with a polymer shell. In addition, the diameters of MPs and Cap-MPs were measured using Image-pro plus 6.0 (Media Cybernetics, Inc., Rockville, MD, USA) as approximately 1.45 ± 0.057 µm and 1.39 ± 0.030 µm, respectively. This result indicated that recombinant Cap was successfully coupled to MPs.

### 2.3. Optimization of Working Conditions

In order to achieve optimal working conditions for the developed CLEIA, important parameters were optimized.

#### 2.3.1. Doses of Recombinant Cap Coupled to MPs

**Figure 3:**
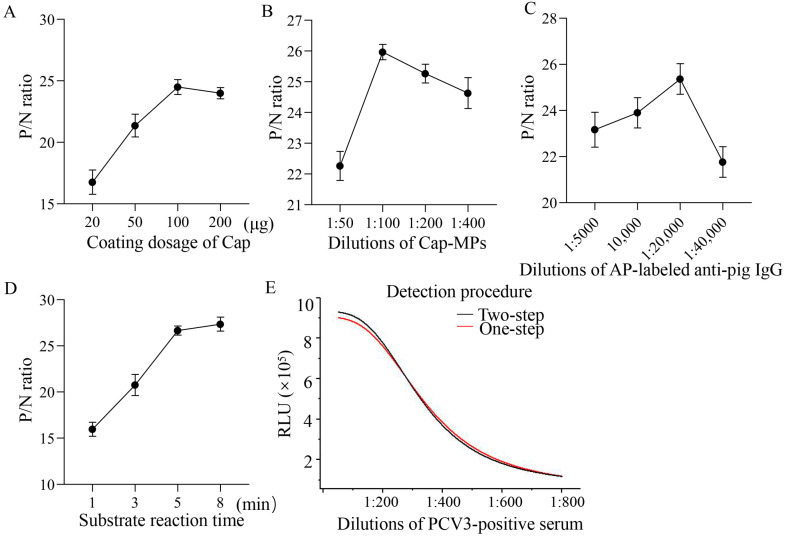
Optimization results for CLEIA. (**A**) Determination of optimal coating dosage of purified Cap. (**B**) Determination of optimal dilution of Cap-MPs. (**C**) Determination of optimal dilution of AP-conjugated goat anti-pig IgG. (**D**) Optimal reaction time (1, 3, 5, 8 min) for substrate. (**E**) Optimal antigen–antibody reaction model, one-step and two-step were assessed, respectively.

In order to obtain a better assay and more economical doses of beads and protein, multiple doses of recombinant Cap were coupled to MPs. As shown in Figure 3A, the P/N ratio increased with increasing doses of Cap in the range of 20 to 100 µg. When the dose was higher than 100 µg, the increase started to slow down, and the dose of 100 µg was used for subsequent assay.

#### 2.3.2. Dilutions of Cap-MPs and the AP-Conjugated Antibody

Antigen-coupled MPs and AP-conjugated antibodies were key parameters influencing sensitivity and specificity. In this study, Cap-MPs and AP-conjugated goat anti-pig IgG were diluted in a series of dilutions. As shown in Figure 3B,C, the ratios of the RLUs of positive and negative sample (P/N ratios) increased with the dilution of Cap-MPs and AP-conjugated goat anti-pig IgG until they reached a maximum. When the dilution of Cap-MPs was 1:100, and the dilution of AP-conjugated goat anti-pig IgG was 1:20,000, their P/N ratios reached maximum values. Therefore, the dilution of Cap-MPs and AP-conjugated goat anti-pig IgG at 1:100 and 1:20,000 were used for further research.

#### 2.3.3. Reaction Time of Substrate Solution

The AP-AMPPD system is a kind of glow type. The luminescence time can last from a few minutes to more than ten minutes, and the optimum detection time should be set at a point where the luminescence signal is relatively stable [18]. The reaction time was set at 1 to 8 min. The P/N ratios were essentially in a steady state as the reaction time was increased. As shown in Figure 3D, the P/N value reached its maximum when the reaction time was 5 min. Therefore, the reaction time for CLEIA is set at 5 min for subsequent assays.

#### 2.3.4. Detection Procedure (One-Step and Two-Step)

In the experiment, one-step and two-step assays were performed under identical conditions. Positive sera were detected at dilutions ranging from 1:100 to 1:1600. As shown in Figure 3E, the R^2^ between RLU values and dilutions of PCV3-positive serum (relative antibody levels) from one-step and two-step assays were 0.99989 and 0.9933, respectively, suggesting that two assays were appropriate. The one-step assay was chosen because it saves at least 10 min.

### 2.4. Determination of Cut-Off Value

**Figure 4:**
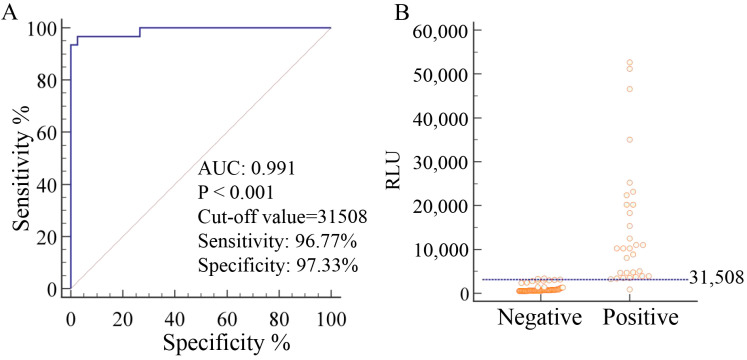
Determination of the cut-off value. The analysis was performed on PCV3-positive serum samples (n = 29) and PCV3-negative serum samples (n = 89) using MedCalc software (Version 19.0.1). (**A**) Dot plot diagram. (**B**) ROC analysis.

Under optimal conditions, a total of 118 samples were detected to determine the cut-off value. According to receiver operating characteristic (ROC) curve analysis, the cut-off value of the developed CLEIA was 31,508, with a diagnostic sensitivity of 96.8% and a diagnostic specificity of 97.3%. The area under the curve (AUC) was 0.991, with 95% confidence (Figure 4A,B). These results indicate high accuracy.

### 2.5. Assessment of Cross-Reactivity of CLEIA

**Figure 5:**
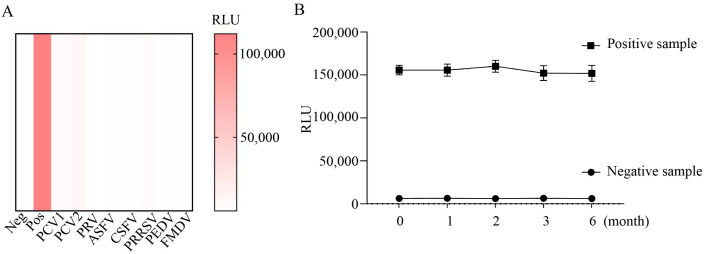
Cross-reactivity and stability test. (**A**) Specificity assay. PCV3-negatitive serum, PCV3-positive serum and PCV1, PCV2, PRV, ASFV, CSFV, PRRSV, PEDV, and FMDV positive serum were measured. Data represent the mean ±SD (standard deviation) from three independent experiments. (**B**) Prepared Cap-MPs were stored with other components at 4 °C for 0, 1, 2, 3, and 6 months. One positive sample and one negative sample were detected.

Cross-reactivity was assessed by testing PCV3-positive serum, negative serum, and serum positive for PCV1, PCV2, CSFV, porcine pseudorabies virus (PRV), African swine fever virus (ASFV), PRRSV, foot-and-mouth disease virus (FMDV), and PEDV. As shown in Figure 5A, only the RLU value of the PCV3-positive serum was above 31,508, indicating that the established CLEIA has a high specificity for the detection of PCV3 Cap-Abs.

### 2.6. Stability Test

All components were stored at 4 °C for 0–6 months. One positive sample and one negative sample were detected at 0, 1, 2, 3, and 6 months, respectively. As shown in Figure 5B, all RLU values of the positive sample were above the cut-off value, with no significant decrease, and those of the negative control were below the cut-off value. These results indicate good stability of the developed CLEIA.

### 2.7. Repeatability Test

To evaluate the reproducibility of the developed CLEIA, we determined six serum samples by intra- and inter-batch reproducibility tests. The intra-batch CV ranged from 1.82% to 4.14% and the inter-batch CV from 3.27% to 8.05% (Table 1), indicating that the developed CLEIA has high reproducibility.

### 2.8. Comparison of CLEIA with Commercial Kit

To evaluate the performance of the developed CLEIA, a comparative study was performed.

Detection rapidity: The proposed CLEIA required only 20 min to automatically complete a sample test, including incubation, immunoreaction, washing, and result generation, whereas traditional ELISA required more than 70 min of tedious operations. Our method also supports between 360 and 480 tests per hour, depending on machine configuration, an advantage that ELISA does not have.

Coincidence rate: To further evaluate the performance of the developed CLEIA, 187 porcine serum samples were tested, including 109 samples from sows and 78 samples from piglets. As shown in Table 2, 103 positive samples and 84 negative samples were detected by the developed CLEIA, and 96 positive samples and 91 negative samples were detected by the commercial kit. The overall agreement rate of the two methods was 95.19%, the positive agreement rate was 92.23%, and the negative agreement rate was up to 98.81%. To correct the agreement for the probability of random coincidence, the Kappa (ĸ) coefficient was calculated, and ĸ was 0.9035, indicating very good agreement. Thus, these results showed that our CLEIA has promising potential for clinical application.

### 2.9. Serological Evidence for PCV3 Infection in South China

**Figure 6:**
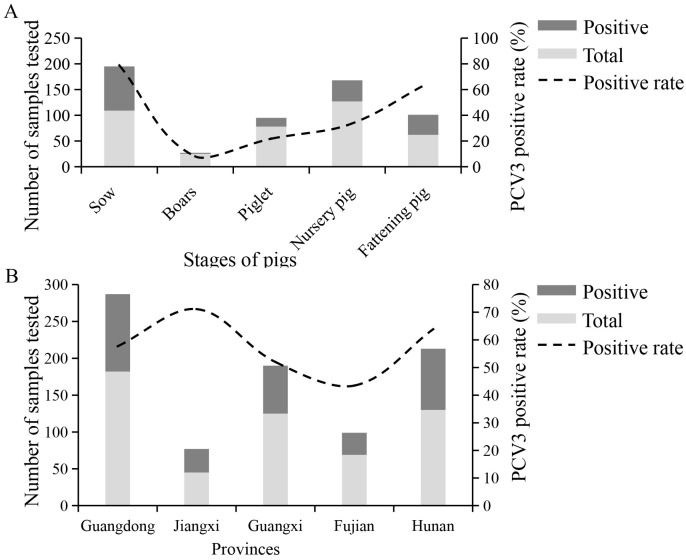
Results of positive rates analysis. (**A**) PCV3 positive rates on farms in different stages of pigs. (**B**) PCV3 positive rates on farms in different provinces.

Based on the results of 551 serum samples, PCV3 has been circulating in South China, as evidenced by the high positive rates of 57.00% (Guangdong), 71.11% (Jiangxi), 52.00% (Guangxi) 43.48% (Fujian), and 63.85% (Hunan). From 2021 to 2023, the prevalence of PCV3 in pig farms in South China was at a high level of 52.68%, 53.33%, and 60.90% (Table 3). The seroprevalence of PCV3 was <50% in boars and piglets, but very high (>50%) in nursery and fattening pigs, and the highest in sows (Figure 6A,B). These results suggest the widespread nature of PCV3 in herds with clinical presentations.

## 3. Discussion

Since 2015, PCV3 has been identified as a major pathogen associated with mummified fetuses aborted from sows with PDNS-like lesions. It is already widespread in many countries, and serves as a potential causative agent [19]. As no vaccine is commercially available, serological testing is a useful method to assess antibody levels for epidemiological surveillance and vaccine development. To date, conventional ELISA is well established and commercially available. However, its time-consuming and labor-intensive operation and high cost (imported kits) are major drawbacks [20]. This study aims to develop a novel CLEIA, consisting of PCV3 Cap-coupled MPs, AP-conjugated secondary antibodies, and an automated chemiluminescence detection instrument, in the hope of achieving the simpler, faster, and more sensitive detection of PCV3 Abs.

CLEIA is widely used in life sciences, environmental management, and food security. However, its use in veterinary diagnostics is limited. Previous studies have shown that CLIAs have been established to detect antibodies to porcine pathogens of ASFV, and porcine parvovirus (PPV) with high sensitivity [21,22]. A multi-protein-based CLEIA showed unique advantages in not only being able to rapidly and accurately discriminate FMDV-infected pigs from vaccinated pigs, but also in effectively avoiding higher false-positive rates caused by non-structural proteins contaminating inactivated vaccines [23]. And a fully automated CLIA has an important application in modern vaccine quality control [24]. Therefore, various types of CLIA are increasingly highlighting its advantages, and gradually accepting and valuing it in the field of veterinary diagnosis.

The newly developed CLEIA was found to be highly sensitive, rapid, and convenient for the detection of PCV3 Cap-Abs. Time to result was generally less than 18 min for a test, compared to at least 70 min required for a commercial kit. The automated procedure avoids tedious steps. It benefits from a suitable detection system. First, this assay used MPs instead of traditional 96-well plates to immobilize Cap. MPs could provide many more active binding sites and allow automated detection [25]. Second, an AP-AMPPD system was used for signal acquisition. Its reaction is fast and sensitive, and luminescence can provide more accurate results in a very short time. It has been used in CLEIAs developed for the detection of antibodies against swine viral pathogens such as ASFV and CSFV, etc. [22,26]. Finally, antibody determination using an automated instrument not only significantly reduces manual operations, such as sample addition, liquid discard, and washing, but also improves detection throughput and consistency [27]. It is worth noting that modern chemiluminescent instruments can detect between 360 and 480 or more samples per hour, depending on chemiluminescent design and instrument performance, which is an advantage that traditional ELISAs do not have.

The new CLEIA was established based on PCV3 Cap. In this study, truncated Cap was successfully expressed and self-assembled into VLPs using baculovirus with good immunogenicity and reactivity. The N-terminal NLS domain of Cap is rich in arginine residues, which may impede the expression of a foreign gene [28], and cause the misfolding of recombinant Cap [29]. Therefore, the removal of NLS has been utilized to improve expression efficiency and stability. Most existing studies have employed this strategy to produce PCV2 or PCV3 Cap [30]. Our system proved to be simpler and more efficient than previous studies using Bac-to-Bac systems. Similar to PCV2 or PCV4 Cap, the prepared Cap can self-assemble into VLPs with a size of approximately 20 nm [11,31]. Previous reports indicated that PCV3 Cap was widely expressed using *E. coli*, and was employed to develop a commercial indirect ELISA kit, which was used in our study [32,33]. Consequently, we also utilized *E. coli* to produce Cap. Although the purified Cap can assemble into VLPs, they were not uniform in size. A properly assembled VLP would mimic surface epitopes of Cap in a correct conformation; thus, a high proportion of correctly assembled VLPs is preferable for the development of serological tests or vaccines. However, the cross-reactivity of Cap between PCV3 and other PCVs needs to be investigated in a timely manner as more PCVs are identified.

The newly developed CLEIA exhibits high specificity and promising potential for clinical use. In order to assess its accuracy and specificity, it was compared to a commercially available kit, revealing a negative concordance rate of 98.81% and an overall concordance rate of 95.19%. Subsequent serological surveys indicated a widespread presence of PCV3 in China, with a high positive rate ranging from 50 to 60% in samples collected from South China between 2021 and 2023. Notably, sows and fattening pigs exhibited PCV3 positive rates exceeding 60%. Prior to 2018, field studies demonstrated a high seroprevalence of PCV3 across all samples, reaching 50%. Seroprevalence in grower-finisher pigs varied from 22% to 80%, while in sows it could reach as high as 96% [34]. Although serological data for the period between 2021 and 2023 was inconclusive, molecular data revealed an average PCV3 prevalence of 31.07% across all provinces from 2018 to 2022 [35]. These findings suggest a significant level of PCV3 circulation in China. Given its pathogenesis and potential for co-infection with other pathogens, PCV3 warrants serious attention.

Automated analytical assays offer the possibility of absolutely quantitative results. The quantification of antibody responses or conversion rates can provide information not only for estimating vaccine responses and duration of protection, but also for improving vaccine immunogenicity, dosage optimization, amount, and time intervals [36]. So, a standard of Cap-Abs is urgently needed to calibrate quantitative analytical methods. In addition, large-scale, continuous assays increase labor costs, and automated assays are a good solution to this challenge. In addition, we are also conducting further studies to reduce detection time and interference from background signals using novel probes, such as acridinium ester (AE), which requires only seconds to complete chemiluminescence reactions compared to 5–10 min for AP or HRP. This ability to perform simultaneous analyses for different indicators [37], which also greatly improves assay efficiency, is an advantage that traditional ELISA does not have.

## 4. Materials and Methods

### 4.1. Serum Samples

Serum samples positive for PCV1, PCV2, PRV, ASFV, PRRSV, CSFV, FMDV serotypes O and PEDV were confirmed and kept in our laboratory [38]. A total of 118 samples, including 89 negative samples and 29 positive samples, were collected and confirmed by an imported and widely used commercial kit (Lot:JS221223, BioStone, Southlake, TX, USA). From 2021 to 2023, a total of 551 clinical samples were collected from South China (Guangdong, Fujian, Jiangxi, Guangxi, and Hunan). These samples were collected from pigs of different ages, including sows, boars, piglets, nursery pigs, and fattening pigs, with suspicion of PCV-associated diseases (PCVAD). All sera were inactivated at 56 °C for 30 min and stored at −80 °C until use.

### 4.2. Expression and Purification of PCV3 Cap Using Baculovirus

The ORF2 gene fragment of PCV3 with nuclear location signal (NLS) deletion from strain GD2016 (GenBank: KY418606) was optimized, synthesized, and subcloned into pQB3 transfer vectors (Bacmid Co., Ltd., Xi’an, China) to generate the recombinant transfer vector pQB2-ORF2. Using FuGENE HD transfection reagent (No E2311, Promega, Beijing, China), the linearized bacmid DNA qBac-IIIG (No10302, Bacmid Co., Ltd., Xi’an, China) and the pQB2-ORF2 were co-transfected into Sf9 cells at 50–60% confluence to generate a recombinant baculovirus. After 5 days of culture at 28 °C, the expression of recombinant Cap was identified by IB using anti-PCV3 Cap monoclonal antibody. Infected cells were then harvested and resuspended in phosphate-buffered saline (PBS, pH 7.4) for protein purification. The soluble Cap was purified using BeaverBeads^TM^ IDA-Nickel kit (No70501-K10, Beaver, Suzhou, China). The purified Cap was dissolved in PBS and analyzed by SDS-PAGE. Its concentration was measured using a BCA protein assay kit (No P0009; Beyotime, Shanghai, China).

To characterize recombinant Cap, TEM was performed as follows: 20 µL of Cap suspension was dropped onto 200 mesh cuprum grids with carbon film (ZB-C1004, ZXBR, Beijing, China) for 3–5 min, then stained with 2% phosphotungstic acid (G1102, Servicebio, Wuhan, China) for 1–2 min, and dried at room temperature. Images were taken with a transmission electron microscope (HT7800, Hitachi, Tokyo City, Japan).

### 4.3. Expression and Purification of Recombinant Cap Using E. coli

The ORF2 gene fragment with NLS deletion was optimized and subcloned into the pColdⅠ vector to generate the recombinant plasmid pCold-ORF2. The recombinant pCold-ORF2 was then transformed into *Escherichia coli* BL21 (DE3) host strain (No CD801-02, Takara, Beijing, China). The monoclonal colony was resuspended and grown in 50 mL of Luria–Bertani (LB) medium containing 100 µg/mL ampicillin at 16 °C with shaking at 250 rpm. When an A_600_ value of 0.6–0.8 was reached, 0.1 mM isopropyl-β-d-thiogalactopyranoside (IPTG) was added, and cultures were grown for a further 16 h at 16 °C. Cells were cooled to 4 °C and harvested by centrifugation at 3500× *g* for 15 min. The soluble Cap was purified and analyzed as described above.

### 4.4. Conjugation of Cap Protein to MPs

Purified Cap was coupled to MPs according to previous studies, with some modifications. Briefly, 1 mg of hydrophilic MPs (MG01, MadeNeW, Changsha, China) was washed and resuspended in 900 μL borate buffer (0.1 M, pH 8.0), followed by the addition of 100 μg purified Cap and 500 μL sodium sulphate (1 M), and incubated at 37 °C for 18 h on a rotator. Then 15 μL BSA (10%) was added and incubated at 37 °C for a further 6 h. After washing with TBS buffer (pH7.4, containing 0.05% Tween20), the conjugated Cap-MPs were dissolved in PBS (pH 7.4, containing 150 mM NaCl, 1% BSA, 0.05% Tween-20, 0.05% ProClin300), and stored at 4 °C until use.

To characterize MPs and Cap-MPs, SEM was performed as follows: 50 uL of MPs or Cap-MPs were dropped onto 200 mesh cuprum grids with carbon film (ZB-C1004, ZXBR, Beijing, China) and dried at room temperature. Conductive metal coating: Samples were attached to metallic stubs using carbon stickers and sputter-coated with gold for 30 s. Images were taken with a scanning electron microscope (MC1000, Hitachi, Tokyo City, Japan). Then, 10 particles from each group were randomly selected and their diameters were measured using Image-pro plus 6.0 by Servicebio (Wuhan, China).

### 4.5. CLEIA Procedure

In this study, an indirect CLEIA was developed using Cap-MPs as carriers and the AP-conjugated secondary antibody complex to catalyze the conversion of chemiluminescent substrate. To detect Cap-Abs, 30 µL of serum sample and 100 µL Cap-MPs were co-added to a test tube (KEYLIGHTS, Shenzhen, China) and incubated at 37 °C for 10 min. After three washes with TBST (Tris buffered saline containing 0.1% Tween-20) for 4 s, 100 µL of AP-conjugated goat anti-pig IgG (Abcam, Cambridge, MA, USA) was added and incubated at 37 °C for 10 min. After three washes with TBST for 4 s, 100 µL of AMPPD was added to measure relative light units (RLU) using an automated instrument (Venus 100H, KEYSMILE, Chongqing, China).

### 4.6. Optimization of Parameters

#### 4.6.1. Optimization of Doses of Coated Antigen

The dose of Cap coupled to MPs was optimized as follows. Purified Cap at 20, 50, 100, and 200 μg was respectively mixed with 1 mg of MPs at 37 °C for 2 h. 100 µL of Cap-MPs at of 1:50 dilution was used to test a positive sample and a negative sample. The RLUs of the positive serum and the negative sera were measured. The P/N ratios were compared.

#### 4.6.2. Optimization of Dilutions of Cap-MPs

100 µL of Cap-MPs at dilutions of 1:50 1:100, 1:200, and 1:400 was used to measure positive and negative samples, and the P/N ratios were calculated.

#### 4.6.3. Optimization of Dilutions of AP-Conjugated Antibody

Dilutions of AP-conjugated goat anti-pig IgG (1 mg/mL) were investigated as follows. Regarding concentration, the conjugated antibody was diluted at 1:5000, 1:10,000, 1:20,000, or 1:40,000. The P/N ratios of different conditions were compared.

#### 4.6.4. Optimization of Reaction Time

The reaction time of the substrate solution was investigated. The reaction time was set at 1, 3, 5, and 8 min. The P/N ratios were compared.

#### 4.6.5. Optimization of Detection Procedure

One-step and two-step models were compared. The two-step procedure was performed as described, as a “CLEIA procedure”. The one-step procedure was performed as follows: 100 µL Cap-MPs, 30 µL serum sample and 100 µL of diluted AP-conjugated goat anti-pig IgG were added together in a test tube and incubated for 10 min at 37 °C. After three washes, 100 µL of substrate was added. The correlation coefficient between the RLU value and dilutions of PCV3-positive serum was compared.

### 4.7. Determination of Cut-Off Value, Diagnostic Sensitivity, and Specificity

A total of 118 serum samples including 29 PCV3 positive and 89 PCV3 negative serum samples were detected to determine the cut-off value and assess the diagnostic sensitivity and specificity. The data were analyzed using ROC curve analysis.

### 4.8. Determination of Specificity

Positive sera for common porcine pathogens, including PCV1, PCV2, PRV, ASFV, FMDV type O, CSFV, PRRSV, and PEDV, were detected to evaluate the cross-reactivity of the developed CLEIA.

### 4.9. Determination of Stability

To determine the stability of the developed CLEIA, all components including prepared Cap-MBs, sample dilution, wash buffer, and substrate were stored in test tubes at 4 °C and assayed at 0, 1, 2, 3, and 6 months, respectively.

### 4.10. Determination of Reproducibility

To test intra-assay reproducibility, two negative samples and four serum positive samples for PCV3 were assayed three times. Three batches of Cap-MPs were used to confirm inter-assay reproducibility. The average value of luminescence (M) and SD were calculated. The coefficient of variation (CV) was calculated as (SD/M) × 100%.

### 4.11. Analysis of Clinical Serum Samples and Comparison of CLEIA with Commercial Kit

In this study, 551 clinical samples were detected by established Cap-based CLEIA to investigate the prevalence of PCV3 infection in China. These field serum samples were collected from different stages of pigs on 35 pig farms in five provinces during 2021–2023. Of these, samples from sows and piglets were further tested using an imported commercial kit (Lot:JS221223, Biostone, Southlake, TX, USA) to compare the rate of consistency.

### 4.12. Statistical Analysis

Statistical analysis was conducted using GraphPad Prism version 8.0 software (San Diego, CA, USA) and MedCalc software. Values are expressed as M ± SD. Statistical significance was set at *p* < 0.05. Cohen’s kappa coefficient was calculated using the formula ĸ = (P0 − Pe)/(1 − Pe), where P0 is the relative observed agreement, and Pe is the hypothetical probability of random agreement.

## 5. Conclusions

In conclusion, our CLEIA has excellent potential to elucidate PCV3 epidemiology. Based on this assay, PCV3 has been widely circulating in South China. Due to its advantages of high sensitivity and specificity, rapidity, wide range, and quantitative detection, the developed CLEIA can provide important technological support for epidemiological surveillance and vaccine development, and has very good prospects for application.

## Figures and Tables

**Table 1:** Results of the repeatability assay.

Sample No.	Intra-Assay		Inter-Assay	
	M ± SD	CV (%)	M ± SD	CV (%)
1	6107 ± 158.11	2.59	6110 ± 200.06	3.27
2	6975 ± 127.22	1.82	6868 ± 285.22	4.15
3	56,298 ± 2339.61	4.14	57,042 ± 3074.56	5.39
4	58,624 ± 1829.07	3.12	59,414 ± 4782.83	8.05
5	170,571 ± 6259.96	3.67	184,555 ± 13,103.41	7.10
6	182,180 ± 4645.59	2.55	193,328 ± 12,004.99	6.21

**Table 2:** Coincidence rate of CLEIA and commercial ELISA kit.

Method		Commercial ELISA			
		No. Positive	No. Negative	Total	Coincidence Rate
CLEIA	No. Positive	95	8	103	92.23% (95/103)
	No. Negative	1	83	84	98.81% (83/84)
	Total	96	91	187	95.19% (95 + 83)/187)

**Table 3:** PCV3 positive rates on farms in 2021–2023.

Year	No. Samples Tested	No. Positive	Positive Rate (%)
2021	112	59	52.68
2022	150	80	53.33
2023	289	176	60.90

## Data Availability

The data presented in this study are available on request from the corresponding author.
